# Periodontal Health Changes During Lower Anterior Crowding Treatment With a Modified Inman Appliance: A Prospective Cohort Study

**DOI:** 10.7759/cureus.78909

**Published:** 2025-02-12

**Authors:** Ziad Mohamad Alhafi, Mohammad Y. Hajeer, Ahmad S. Burhan, Mowaffak A. Ajaj, Samer T. Jaber, Alaa Oudah Ali Almusawi

**Affiliations:** 1 Department of Orthodontics, Faculty of Dentistry, University of Damascus, Damascus, SYR; 2 Department of Orthodontics, Faculty of Dentistry, Al-Wataniya Private University, Hama, SYR; 3 Department of Orthodontics, Faculty of Dentistry, University of Al-Knooz, Basrah, IRQ

**Keywords:** crowding relief, gingival index, lower anterior teeth crowding, lower crowding, malocclusion with crowding, papillary bleeding index, periodontal health status, plaque index, the modified inman appliance

## Abstract

Objective

This study investigated periodontal health changes in adult patients with mild crowding, undergoing treatment with a modified Inman appliance.

Materials and methods

This prospective cohort study involved 16 malocclusion patients (4 males and 12 females; mean age 21.94 ± 2.26 years) with mild crowding. Participants were treated using a modified Inman appliance. Plaque Index (PI), Gingival Index (GI), and Papillary Bleeding Index (PBI) were measured at four assessment times: before treatment (T0), two weeks (T1), one month (T2), and two months (T3) after appliance placement.

Results

All three indices (PI, GI, and PBI) exhibited significant differences across the assessment times (p < 0.001). Pairwise comparisons revealed significant differences in most cases (p ≤ 0.0125), with exceptions primarily observed in comparisons involving T2-T3.

Conclusions

Periodontal health can be negatively affected during orthodontic treatment due to the difficulty of maintaining optimal oral hygiene with the modified Inman appliance. This can lead to increased plaque accumulation and a subsequent rise in periodontal indices. However, these changes typically remain within acceptable limits. Patient-specific factors, such as oral hygiene practices, significantly influence the extent of these changes, emphasizing the importance of diligent oral care throughout treatment.

## Introduction

Fixed orthodontic treatment, while highly effective in correcting misaligned teeth, can sometimes lead to gingival-related issues [1,2]. These problems typically arise from increased plaque buildup and difficulty maintaining optimal oral hygiene during treatment [3-5]. Gingivitis, a mild form of periodontal disease characterized by inflammation and redness of the gingiva, can be exacerbated by poor oral hygiene, particularly during orthodontic treatment, as the presence of braces can hinder effective plaque and tartar removal [6,7]. If gingivitis is left untreated, it can progress to periodontitis, a more severe form of periodontal disease that can result in bone loss and tooth loss [5]. Braces can sometimes cause the gingiva to recede, exposing more of the tooth's root. This can make teeth more sensitive and susceptible to decay [8].

In some cases, braces can lead to an overgrowth of gingival tissue. This is often caused by poor oral hygiene or an allergic reaction to the materials used in the braces [7]. To minimize the risk of periodontal problems during orthodontic treatment, it is essential to maintain acceptable oral hygiene [9]. This includes brushing teeth after every meal, flossing daily, and using an interdental cleaner to remove food particles trapped around the braces [10]. Regular dental check-ups are also crucial to monitor the health of gingiva and ensure that any issues are addressed promptly [11].

The increasing focus on aesthetic appearance in society has recently led to a greater desire among adults to solve minor dental problems such as mild crowding [12]. However, instead of choosing traditional orthodontic appliances due to the unaesthetic metallic appearance of wires and brackets, they increasingly tend toward restorative procedures that may have the potential to damage tooth tissues [13]. As a result, there is an increasing preference for invisible orthodontic methods, especially among adults [14,15]. Consequently, many techniques and materials have been created in clinical practice to alleviate these limitations, such as ceramic brackets, lingual orthodontic techniques, and clear aligners, but these options tend to be very expensive [12,15,16]. The spring aligner, also known as the spring retainer, was introduced by Barrer in 1975 as a removable appliance for aligning incisors [17,18]. It has been used for over 25 years. However, its activation was limited and was only used to correct minor problems after orthodontic treatment and for retention [17]. In 2001, Donn Inman modified the traditional spring retainer and invented the Inman Aligner, a removable appliance for multiple orthodontic purposes [17]. This new appliance relied on super-elastic open coil springs to apply light and constant forces to the anterior teeth' labial and lingual surfaces [19]. Subsequently, the Inman appliance was modified, resulting in a fixed type that applies continuous light forces without needing patient compliance [19,20].

The efficacy and feasibility of this technique have been evaluated [19,20], but the impact on periodontal tissues remains unexplored. Therefore, this prospective cohort study aims to fill this knowledge gap by investigating periodontal indices during treatment with the modified Inman appliance.

## Materials and methods

Study design

A cohort study was conducted to evaluate the impact of orthodontic treatment using a modified Inman appliance on periodontal indices. Patients were monitored, and periodontal parameters were measured at four assessment intervals: pre-treatment, two weeks, one month, and two months post-treatment initiation. The study protocol was approved by the Medical Research Ethics Council of the University of Damascus (approval number: DN-01062022-3) and received funding from the University of Damascus (reference number: 501100020595).

Sample size estimation

The sample size was determined using Minitab® 20.3 software (Minitab LLC, State College, PA, USA) at an alpha level of 0.05 and a 95% confidence interval. The focus of the current study was the change in the Gingival Index (GI). According to Alsino et al., the standard deviation for this variable was 0.095 [21]. The smallest clinically important difference that could be detected was a change of 0.1 in the GI. Using a paired sample t-test, the required sample size was 14 patients. The number of patients was increased to 16 to account for a potential 10% dropout rate among participants.

Patient recruitment and inclusion criteria

This study involved 16 participants (12 females and 4 men) who were treated between January and September 2023 at the Department of Orthodontics, Faculty of Dentistry, University of Damascus, Damascus, Syria. The inclusion criteria were as follows: (1) mild crowding <4 mm, (2) normal vertical growth pattern, (3) skeletal class I malocclusion (ANB = 2-4), (4) patients within an age range of 18-25 years. The criteria for exclusion were as follows: (1) history of orthodontic therapy, (2) severe skeletal discrepancy, (3) bimaxillary protrusion, (4) bad oral hygiene (Plaque Index, or PI >1). Before participating, each patient received an information sheet outlining the study's purpose and procedures. Following this, all patients provided written informed consent to participate in the research.

Clinical procedures

**Figure 1:**
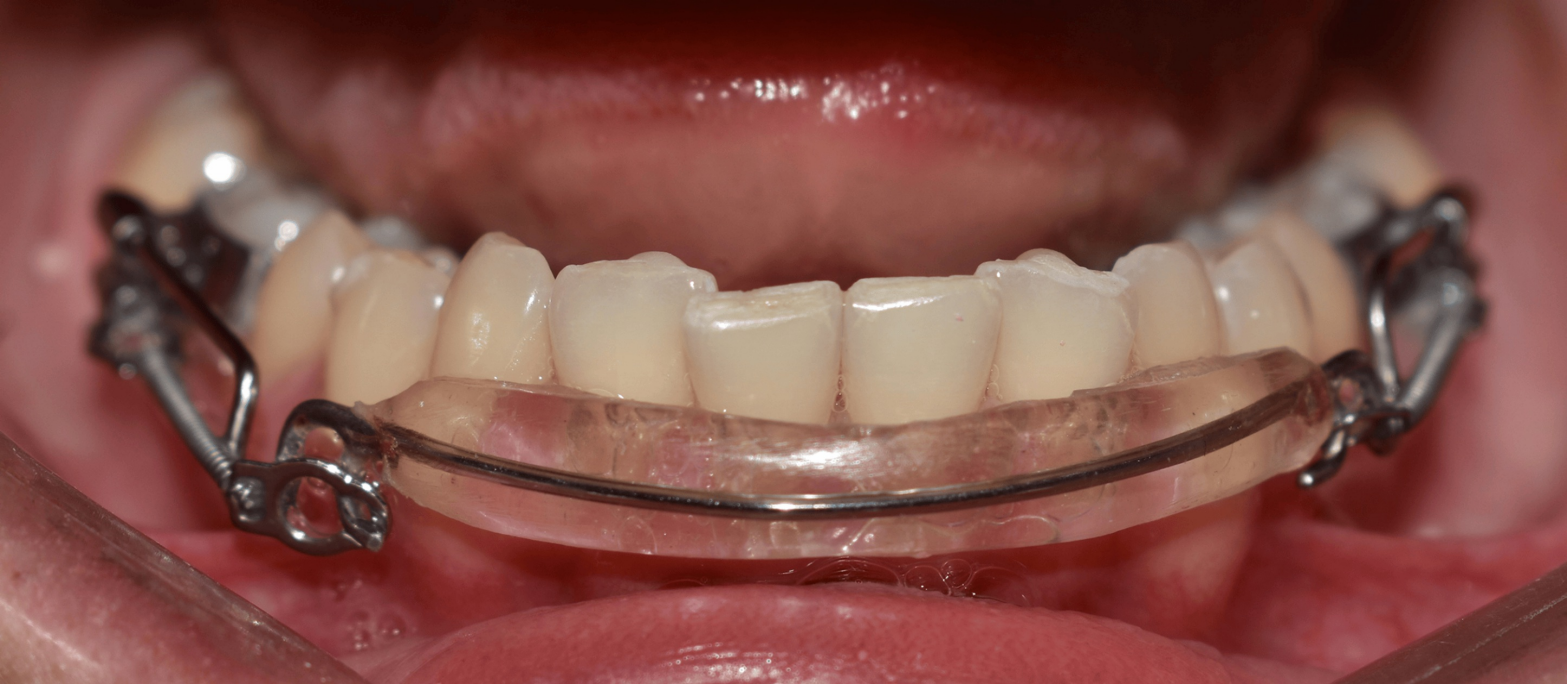
The Inman appliance inserted in the lower dental arch.

Sixteen patients received orthodontic treatment using a modified Inman appliance. This appliance utilizes labial and lingual pads that contact the middle portion of the front teeth. The appliance was designed based on a virtual treatment plan created using the Blue Sky Plan software (version 4.7; Blue Sky Bio, Libertyville, IL, USA). This virtual model was then 3D printed and used as a guide for fabricating the actual appliance. The Inman appliance was placed in the lower jaw. The built-in nickel-titanium springs were activated to apply balanced forces to the teeth, guiding them into the desired alignment. An intraoral orthodontic force gauge was used to measure and adjust the force applied during the procedure to a maximum of 80 grams per side, according to the individual requirements of each case (Figure 1).

Before starting treatment, interproximal reduction (IPR) was performed between the canines to create the necessary space for tooth alignment. Double-sided metal strips were used to carefully reduce the contact points between the teeth. The amount of IPR varied depending on the individual needs of each patient. Treatment was successful when the final tooth alignment met the standards outlined in the American Board of Orthodontics Objective Grading System (ABO-OGS). Patients were seen every two weeks to monitor treatment progress and adjust the spring forces as needed.

Outcomes measures

Before orthodontic treatment began (T0), the following periodontal parameters were assessed: PI, GI, and Papillary Bleeding Index (PBI). These periodontal parameters were then re-evaluated at four different time points: two weeks (T1), one month (T2), and two months (T3), post-treatment initiation.

To assess the PI, a dental explorer and mouth mirror were used to examine the thickness of plaque on the buccal, lingual, mesiobuccal, and distobuccal surfaces of the teeth. The plaque thickness was evaluated after each tooth surface was thoroughly dried with air. The PI for each patient was calculated by averaging the PI values obtained for all six lower teeth [22]. The GI was determined using a millimeter-calibrated periodontal probe (William's probe) and a mouth mirror. The severity of gingivitis was assessed at four sites: the mesiobuccal papilla, the distobuccal papilla, the buccal gingival margin, and the lingual gingival margin. The GI for each patient was calculated by averaging the gingivitis scores obtained for all six lower teeth [22]. The PBI was assessed using a William's probe and a mouth mirror. The severity of bleeding on the papillae's mesial and distal aspects was evaluated. The PBI for each patient was calculated by averaging the bleeding scores obtained for all six lower teeth [23].

Statistical analysis

All statistical analyses were performed using IBM SPSS Statistics for Windows, version 26 (released 2019; IBM Corp., Armonk, NY, USA). A significance level of 0.05 and a 95% confidence interval were used to evaluate the results. The Shapiro-Wilk test was conducted to assess the normality of the data distribution. A repeated measures analysis of variance (ANOVA) test was utilized to identify statistically significant changes within each group over the study period. The paired sample t-test was used for pairwise comparisons. According to the Bonferroni correction, the results were considered significant if the p-value ≤0.0125.

## Results

Baseline sample characteristics

This study involved 16 participants (4 males and 12 females) with an average age of 21.94 ± 2.26 years. All participants successfully completed the study with no dropouts. Table 1 provides a summary of the participants' baseline demographic.

**Table 1:** Baseline sample characteristics (age and gender). SD: standard deviation

Variable		Study sample (n = 16)
Age (in years)	Mean ± SD	21.94 ± 2.26
Gender	Male	4 (25%)
	Female	12 (75%)

Main findings

The descriptive statistics for all periodontal indices measured at all time points are shown in Table 2.

**Table 2:** Descriptive statistics for the values of the periodontal indices at the four assessment times. PI: plaque index; GI: gingival index; PBI: papillary bleeding index; SD: standard deviation; Q1: first quartile; Q3: third quartile; Min: minimum; Max; maximum; T0: before treatment; T1: after 2 weeks; T2: after one month; T3: after 2 months

Variable	Time point	Study sample (n = 16)				
		Mean	SD	Median (Q1-Q3)	Min	Max
PI	T0	0.55	0.22	0.54 (0.35-0.73)	0	1
	T1	0.84	0.32	0.83 (0.68-1)	0	2
	T2	1.16	0.54	1 (0.75-1.58)	0	2
	T3	1.22	0.46	1.10 (0.83-1.69)	1	2
GI	T0	0.36	0.22	0.33 (0.17-0.48)	0	1
	T1	0.75	0.42	0.75 (0.44-0.83)	0	2
	T2	0.90	0.43	0.83 (0.68-1)	0	2
	T3	1.04	0.45	0.92 (0.75-1.33)	1	2
PBI	T0	0.48	0.36	0.49 (0.09-0.65)	0	1
	T1	0.66	0.43	0.57 (0.38-0.91)	0	2
	T2	0.98	0.44	0.90 (0.65-1.52)	0	2
	T3	1.20	0.43	1.14 (0.79-1.57)	1	2

Regarding PI scores, there was a significant difference between the various assessment times (p < 0.001). Pairwise comparisons showed a significant difference in all pairwise comparisons (p ≤ 0.0125), except for T2-T3 (p = 0.155; Table 3).

**Table 3:** Results of changes in the values of the periodontal indices within the study sample. † - Employing repeated measures ANOVA test; ‡ - Employing paired sample t-test; * - p < 0.0125 (according to Bonferroni’s correction) PI: plaque index; GI: gingival index; PBI: papillary bleeding index; CI: confidence interval; SD: standard deviation; T0: before treatment; T1: after 2 weeks; T2: after one month; T3: after 2 months; ANOVA: analysis of variance

Variable	Time point	Study sample (n = 16)						
		Mean (SD)	p-value^†^	Pairwise comparisons				
				Time	Mean difference	95% CI of the difference		p-value^‡^
						Lower	Upper	
PI	T0	0.55 (0.22)	<0.001*	T0-T1	-0.29	-0.14	-4.16	0.001*
	T1	0.84 (0.32)		T1-T2	-0.32	-0.14	-3.90	0.001*
	T2	1.16 (0.54)		T2-T3	-0.06	-0.02	-1.49	0.155
	T3	1.22 (0.46)		T0-T3	-0.67	-0.45	-6.31	<0.001*
GI	T0	0.36 (0.22)	<0.001*	T0-T1	-0.38	-0.54	-0.21	<0.001*
	T1	0.75 (0.42)		T1-T2	-0.15	-0.34	-0.03	0.107
	T2	0.90 (0.43)		T2-T3	-0.13	-0.30	-0.02	0.091
	T3	1.04 (0.45)		T0-T3	-0.67	-0.90	-0.44	<0.001*
PBI	T0	0.48 (0.36)	<0.001*	T0-T1	-0.18	-0.29	-0.07	0.002*
	T1	0.66 (0.43)		T1-T2	-0.31	-0.42	-0.19	<0.001*
	T2	0.98 (0.44)		T2-T3	-0.22	-0.39	-0.05	0.014
	T3	1.20 (0.43)		T0-T3	-0.72	-0.93	-0.50	<0.001*

When studying the GI, there was a significant difference between the various assessment times (p < 0.001). Pairwise comparisons showed a significant difference in all conducted comparisons (p ≤ 0.0125), except for T1-T2 and T2-T3 (p = 0.107 and p = 0.091, respectively). The results of the study of the PBI showed a significant difference between the various assessment times (p < 0.001). Pairwise comparisons showed a significant difference in all conducted comparisons (p ≤ 0.0125), except for T2-T3 (p = 0.014).

## Discussion

As far as we know, this study is the first to investigate the impact of treating mild crowding with a modified Inman appliance on periodontal tissue health. To minimize the risk of overlooking critical developments, assessments were performed every fortnight during the course of treatment to monitor progress meticulously. The majority of participants in this study were women, which aligns with previous studies demonstrating a higher prevalence of orthodontic treatment among adult women [24].

Digital dental scanning was employed to acquire a digital model, a technique recognized for its accuracy and proven efficacy. Furthermore, the device was fabricated using a 3D-printed resin model, a method demonstrated in previous studies to yield clinically acceptable outcomes [25,26]. Nickel-titanium springs were activated with an 80 g force on each side, as individual teeth typically require a force between 35 g and 60 g to induce tipping. Specifically, a 40 g force was applied to each incisor, resulting in a total force of 160 g (80 g per side) [19]. A fixed appliance was utilized in this study to ensure accurate and reliable results. This eliminates the reliance on patient compliance, a critical factor for achieving optimal treatment outcomes with removable appliances, as established by previous research [27,28].

A notable trend observed throughout the treatment period was a progressive increase in the values of all three indices, with statistically significant differences evident between each assessment time point. This gradual elevation culminated in the highest values recorded at the final evaluation, conducted two months after treatment initiation. While this increase occurred, it is crucial to emphasize that these values remained within clinically acceptable limits, indicating that the observed changes did not pose a significant threat to periodontal health [6].

This observed increase in periodontal indices can be attributed to several factors. The modified Inman appliance significantly challenges maintaining optimal oral hygiene [3]. The intricate design of these appliances creates numerous crevices and hard-to-reach areas, hindering the effective removal of plaque by conventional brushing techniques [14]. This difficulty in achieving thorough oral hygiene leads to an accumulation of plaque, which can contribute to the development of gingival inflammation and other periodontal issues [4,5]. These findings align with the conclusions of previous research investigations, which have consistently demonstrated a rise in periodontal indices during orthodontic treatment periods. This phenomenon is primarily attributed to the increased difficulty in maintaining adequate oral hygiene in the presence of any fixed appliances [29,30]. However, it is important to note that this increase in periodontal indices is generally a temporary phenomenon, upon removing orthodontic appliances, diligent oral hygiene practices typically significantly improve periodontal health parameters [31,32].

Furthermore, individual patient factors play a crucial role in determining the extent of these changes. Variations in personal oral hygiene practices, such as brushing frequency, duration, and technique, significantly influence plaque accumulation and subsequent impact on periodontal tissues [31]. Patients with meticulous oral hygiene routines are generally better equipped to maintain periodontal health during orthodontic treatment and experience a more rapid recovery post-treatment [33].

Limitations

The primary limitation of the current study is that the evaluation of periodontal indices was restricted to the mandibular arch due to the application of the modified Inman appliance only on the lower teeth. Furthermore, a comparison between the modified Inman appliance and other orthodontic appliances should be conducted to determine which one exerts the least impact on periodontal health, in treating this type of crowding.

## Conclusions

Periodontal indices, such as PI and GI, gradually increased throughout orthodontic treatment, peaking at the final assessment. While this increase was observed, the values remained within clinically acceptable limits, indicating that the changes were not severe enough to warrant immediate intervention. This rise in periodontal indices is likely due to the inherent difficulties patients experience when maintaining effective oral hygiene with a modified Inman appliance, which can lead to increased plaque accumulation and a subsequent predisposition to gingival inflammation.
